# Effectiveness of education intervention of tuberculosis treatment adherence in Khartoum State: A study protocol for a randomized control trial

**DOI:** 10.1371/journal.pone.0277888

**Published:** 2022-11-28

**Authors:** Khalda Mohamed Khamis, Hayati Kadir Shahar, Rosliza Abdul Manaf, Hamdan Mustafa Hamdan

**Affiliations:** 1 Faculty of Medicine & Health Sciences, Department of Community Health, University Putra Malaysia, Serdang, Malaysia; 2 Federal Ministry of Health, Disease Control Department, Khartoum, Sudan; Prince Sattam Bin Abdulaziz University, College of Applied Medical Sciences, SAUDI ARABIA

## Abstract

**Background:**

Treatment failure and disease relapse among tuberculosis (TB) patients are commonly caused by non-adherence. It can lead to prolonged infection, increased transmission, drug resistance, and loss of life. Even though the causative microorganism of TB has been identified for more than a century, the disease is still a substantial public health problem worldwide. This research aims to devise, implement, and assess an educational intervention to improve adherence to TB treatment.

**Methods and findings:**

A randomised clinical trial involving 146 Sudanese TB patients will be conducted at the Abu Anga hospital in Khartoum. The participants will be randomly assigned to the intervention and control groups. A 2-hour session will be offered to the intervention group in a one-day TB educational intervention course. The same educational materials will also be provided to the control group after the randomised controlled trial (RCT). Data will be collected at baseline, one month, and four months after the intervention. The primary outcome of interest is TB treatment adherence, while secondary outcomes include quality of life score, tuberculosis knowledge, and health belief domains. Generalised estimating equations (GEE) in SPSS software version 25.0 will be utilised to evaluate the changes over time.

**Conclusions:**

This trial will provide information that could be used in improving TB control strategies to achieve better results in the adherence of healthcare services to the norms of the National Program and patient adherence to the disease treatment and cure.

**Trial registration:**

This study is registered at TCTR: (TCTR20210607006).

## Introduction

Tuberculosis (TB) is one of the top ten leading causes of fatalities globally. In 2017, TB was the identified cause of 1.6 million fatalities, particularly in developing countries. It also surpassed HIV/AIDS as the primary cause of death among single infectious agents [1]. TB continues to afflict millions of people annually. World Health Organisation (WHO) estimated that around 10 million people contracted TB in 2020; of these, 5.6 million were men, 3.3 million were women, and 1.1 million were children [2]. The national incidence rate of TB varies from fewer than 5 to above 500 cases of new and relapsing patients for every 100 000 people annually. In 2019, less than half of 54 countries recorded a low TB incidence rate [3]. TB remains a worldwide public health concern as the Tubercle bacillus is likely to have infected one-third of the global population. These people either suffer from the disease or are at risk of progressing to active disease status [4]. Even though the causative bacteria of TB had been identified more than a century ago and effective management strategies, including anti-TB drugs and vaccines, are available, it remains a substantial public health concern worldwide [5]. Treatment failure and disease relapse are commonly caused by non-adherence to treatment, resulting in higher infection rates, transmission, drug resistance, and deaths. A vital component of the WHO-recommended anti-TB strategy is Directly Observed Therapy (DOT). TB patients must take their medication under direct observation of health workers or family members [6]. This strategy is effective in improving treatment adherence. Nevertheless, the coverage rate of DOT is still low in some resource-poor countries and thus fails to improve treatment adherence in those countries [7].

Furthermore, this method requires a substantial investment of time from health workers or commitment from family members. Besides, it can be a financial burden and discourages patient autonomy [8]. As a result of these factors, DOT implementation has been largely restricted. A Chinese study discovered that DOT was only practised by 14% of patients, and worse still, the follow-up loss amounted to 28% [7]. Therefore, an innovative approach needs to be devised to innovate TB therapy. Poor adherence to treatment and failure to follow up raise mortality, morbidity, and the risk of drug resistance development, which can result in the prolonged spread of TB [9].

In Sudan, civil war in certain areas and people’s displacement while searching for a better life are among the various factors currently faced by Sudan, which may lead to the increase in the occurrence and spread of many health hazardous diseases, among them are TB infection and TB treatment adherence [10]. Sudan has adopted the DOTS strategy since 1993. However, failure to adhere to TB treatment continued among some patients, eventually resulting in their default of the treatment before completing it, despite health authorities’ efforts to address this issue [11]. To improve the treatment adherence level, various intervention methods have been designed and implemented across the world [12–14]. As recommended in the WHO End TB Strategy 2017, a set of novel interventions can be employed to raise adherence, such as sending text messages to cell phones, applying digital monitors to keep track of pill containers and medication consumption, as well as providing supportive treatment supervision by treatment partners [15].

In the literature, one of the methods that have been proven to have a positive effect on TB treatment compliance is an intervention based on the enhanced TB adherence model with a focus on empowering both patients and professionals [16]. Various theories and models have been implemented to increase adherence to TB treatment. For example, the Health Belief Model (HBM) is commonly applied as the theoretical framework of many educational interventions. Studies have proven the effectiveness of HBM in improving adherence to TB treatment [17,18]. Similarly, many studies that used educational intervention to improve TB treatment adherence among TB patients have been carried out globally [19,20].

Generally, studies on TB-focused interventions are scarce among newly diagnosed patients, especially in Sudan. Therefore, this study protocol aims to outline the development and implementation of an HBM-based educational intervention in an RCT as a means to educate TB patients in Khartoum, Sudan. Apart from that, this study also assesses the impact in terms of their TB knowledge, health beliefs, and quality of life (QOL). The study hypothesises that at the 1- and 4-month assessments, TB patients in the intervention group will show a raised score in the following areas:

- TB treatment adherence level.
- TB knowledge scores.
- TB adherence to health beliefs (perceived susceptibility, perceived seriousness, perceived benefits, perceived barriers, confidence, and health motivation).
- QOL.

This research aims to devise, implement, and assess an educational intervention to improve adherence to TB treatment among TB patients in Sudan. We hypothesise that an educational module intervention based on the HBM will improve tuberculosis treatment adherence level, health beliefs, QOL, and knowledge among TB patients in Sudan.

## Materials and methods

### Study design

**Fig 1:**
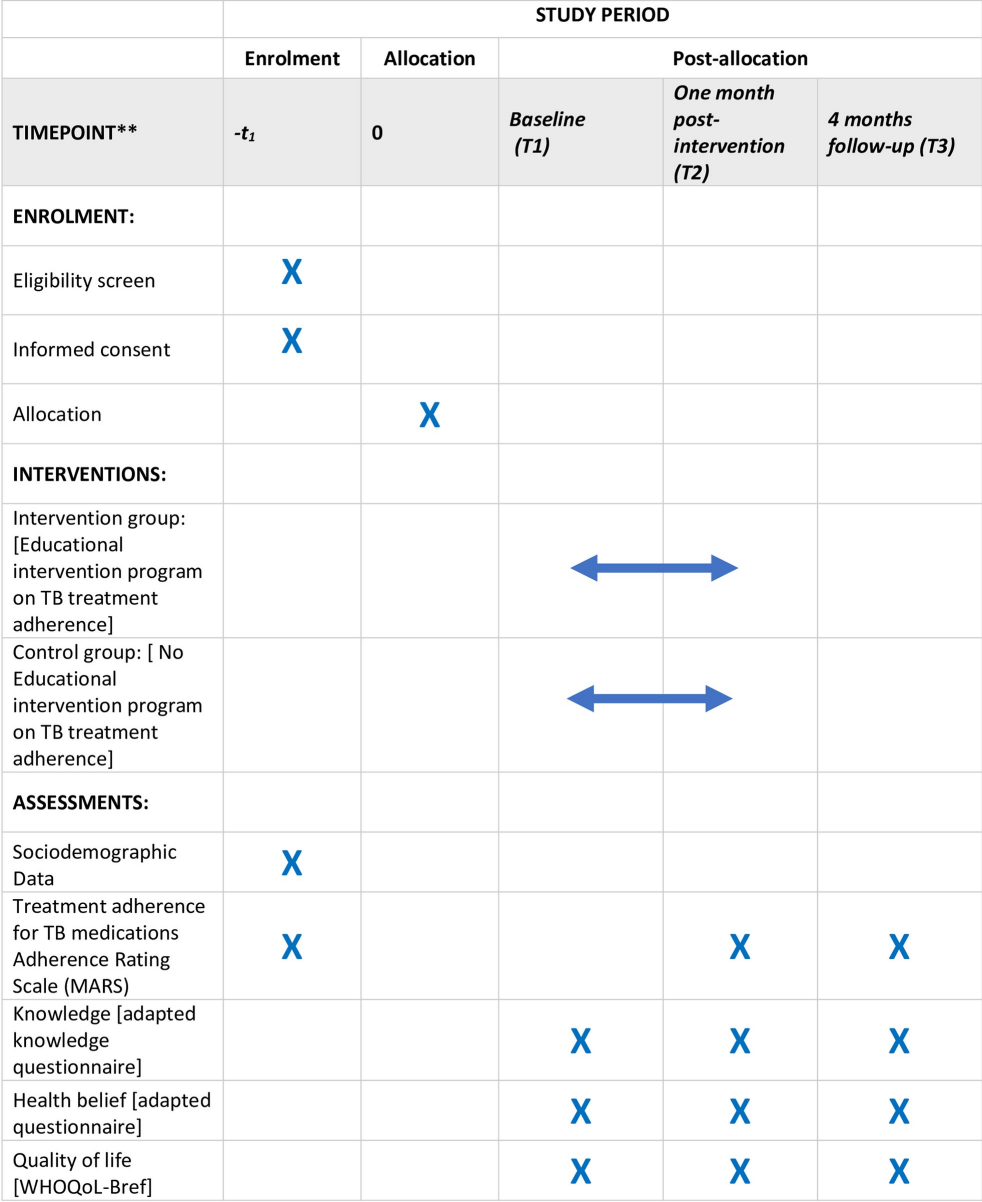
SPIRIT schedule of enrolment, interventions, and assessments [21].

This randomised controlled trial (RCT) will be conducted in a parallel approach using one control group and one intervention group. The group members will include patients diagnosed with pulmonary TB based on the National Treatment Guideline under the Directly Observed Therapy (DOT) strategy. The participants will not be aware of the group assignments (single-blind). The educational material will be given to the intervention group after baseline data collection, while the control group will receive the educational material at the end of the study. Assessments will be carried out at baseline, one month, and four-month post-intervention, as shown in Fig 1.

### Conceptual model

**Fig 2:**
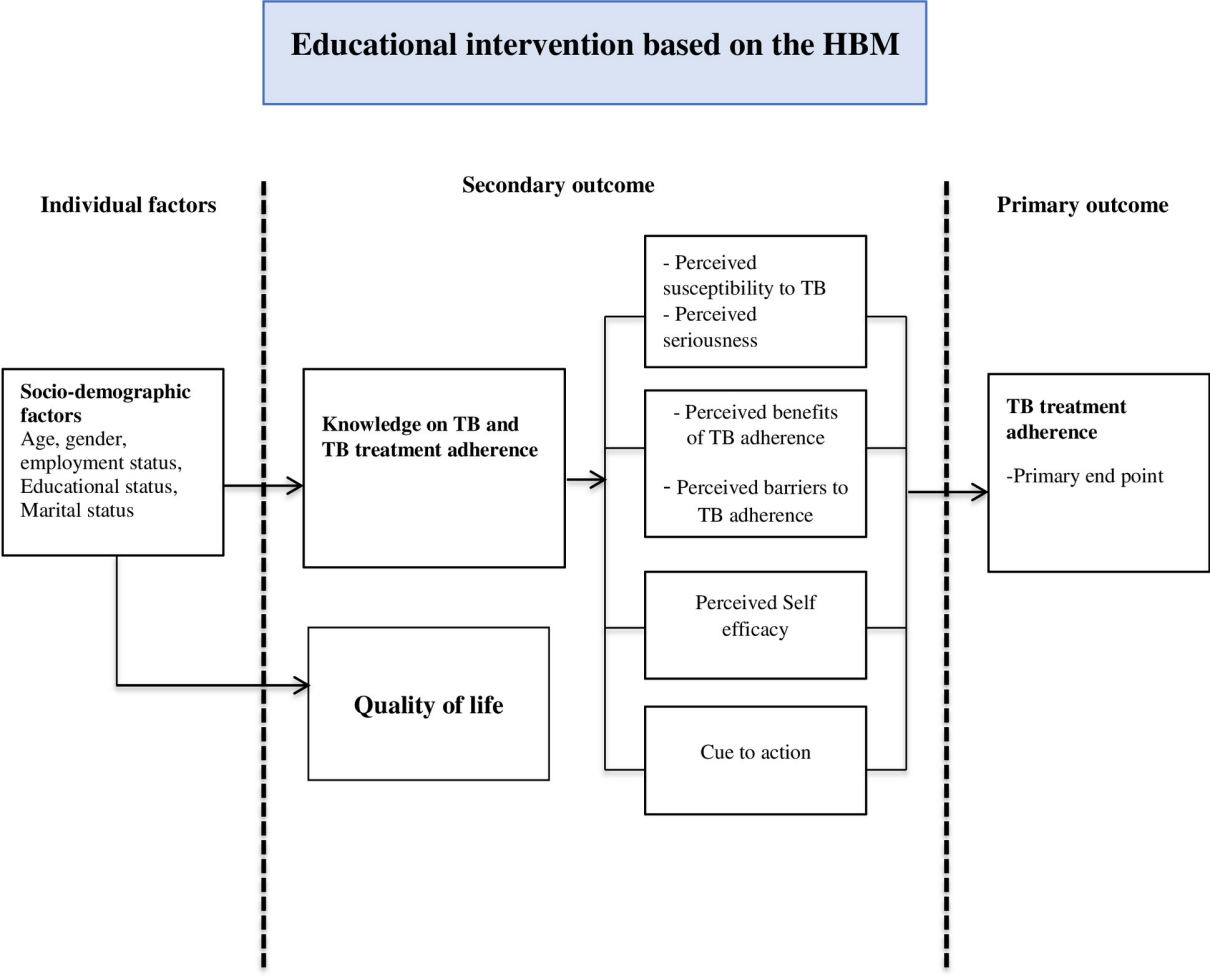
The conceptual model was adapted from the HBM [22].

This trial is grounded on the Health Belief Model (HBM), which focuses on the assessment of an individual’s health behaviour performed by examining perceptions and attitudes that someone may have towards disease and the negative outcomes of certain actions [21]. Fig 2 below depicts the conceptual model adapted from the health belief model.

### Study setting

The study will be based at the Abu Anga regional referral hospital in Omdurman city, Khartoum state, Sudan, which acts as Sudan’s primary TB specialist hospital. All cases of suspected resistant TB are sent to this facility when confirmation is needed. The hospital also provides healthcare services to the people of Khartoum and neighbouring states and cares for suspected TB cases.

### Study participants

Permission will be sought from the hospital authority to allow the research team to collect the information from TB patients. The team will meet the patients during their normal follow-up to introduce the study. The subject will be an individual Sudanese patient with TB aged 18 years and above who were randomly selected from public hospitals and who fulfilled the inclusion and exclusion criteria during the study.

### Eligibility criteria

The inclusion criteria are (1) Patients on a full TB treatment course for 1 or 2 months before the study date; (2) TB patients who can give informed consent physically and mentally and who can abide by the provided intervention with no difficulty or needing assistance; (3) Patients who can commit to the entire course of the study; (4) Patients aged 18 years or older.

The exclusion criteria are (1) Patients with a history of severe and uncontrolled psychiatric disorders; (2) Patients who are unable to read and write in Arabic; (3) TB patients not on a full TB treatment course.

### Ethics approval and informed consent

Ethical clearance for the RCT has been obtained from “Jawatankuasa Etika Universiti Untuk Penyelidikan Melibatkan Manusia” (JKEUPM) at Universiti Putra Malaysia [Ref No UPM/TNCPI/RMC/JKEUPM/(FPSK-P143]. Permission has also been obtained from the Abu Anga hospital in Sudan. The RCT will follow the ethical criteria throughout its entire procedure. Participants will be assured that their participation in the study is voluntary and that they have the full right to withdraw from the study at any time. Each participant must sign a participant consent form before the study. Data collection will be done confidentially and anonymously.

### Sample size

The sample size is calculated using the standard sample size estimate for an individually randomised design. Sample size will be determined according to the primary dependent variable (TB treatment adherence). The formula to calculate the difference between two population proportions will be used (power = 0.80, alpha = 0.05 two‐sided, P_1_ = estimated proportion (larger) P1 = 0.82, P_2_ = estimated proportion (smaller) P2 = 0.69) [22].


$$N=\frac{\{1.96\sqrt{2\times 0.33(1-0.33)}+0.842\sqrt{0.82(1-0.82)+o69(1-0.69)}{\}}^{2}}{{\left(0.82-0.69\right)}^{2}}.$$

N = 171 for each group.

By taking into account a 10% attrition rate, a total sample size of 188 (171 + 17 = 188) participants will be required for each group, giving a total sample size of 376 participants.

### Sampling method

TB patients under the DOTS strategy who fulfil the inclusion and exclusion criteria will be recruited and randomised into the control and intervention groups. The participants will be selected using a simple random sampling technique.

### Data collection

#### Cluster randomized trial flow

*Participant recruitment*. The researcher will inform the respiratory specialist and medical doctors in tuberculosis (TB) clinics about the study objectives and the eligibility criteria to facilitate patient selection for the randomised controlled trial (RCT). After the TB patients complete their appointment with the physician in the respiratory clinic, the nurse will direct the TB patients who fulfil the inclusion criteria to the researcher. The researcher then explains the purpose and benefits of this study. TB patients who agree to join will be asked to complete a written consent form to the relevant authority in Abu Anga hospital before they are asked to answer the questionnaire. After obtaining the baseline assessment, a nurse in the TB clinic will open a sealed envelope to assign the participants to either the intervention or the control groups. Subsequently, the intervention group will be given the TB educational intervention. For the post-intervention and follow‐up assessments, the participants will be asked to fill in the same questionnaire without the section on personal information.

**Fig 3:**
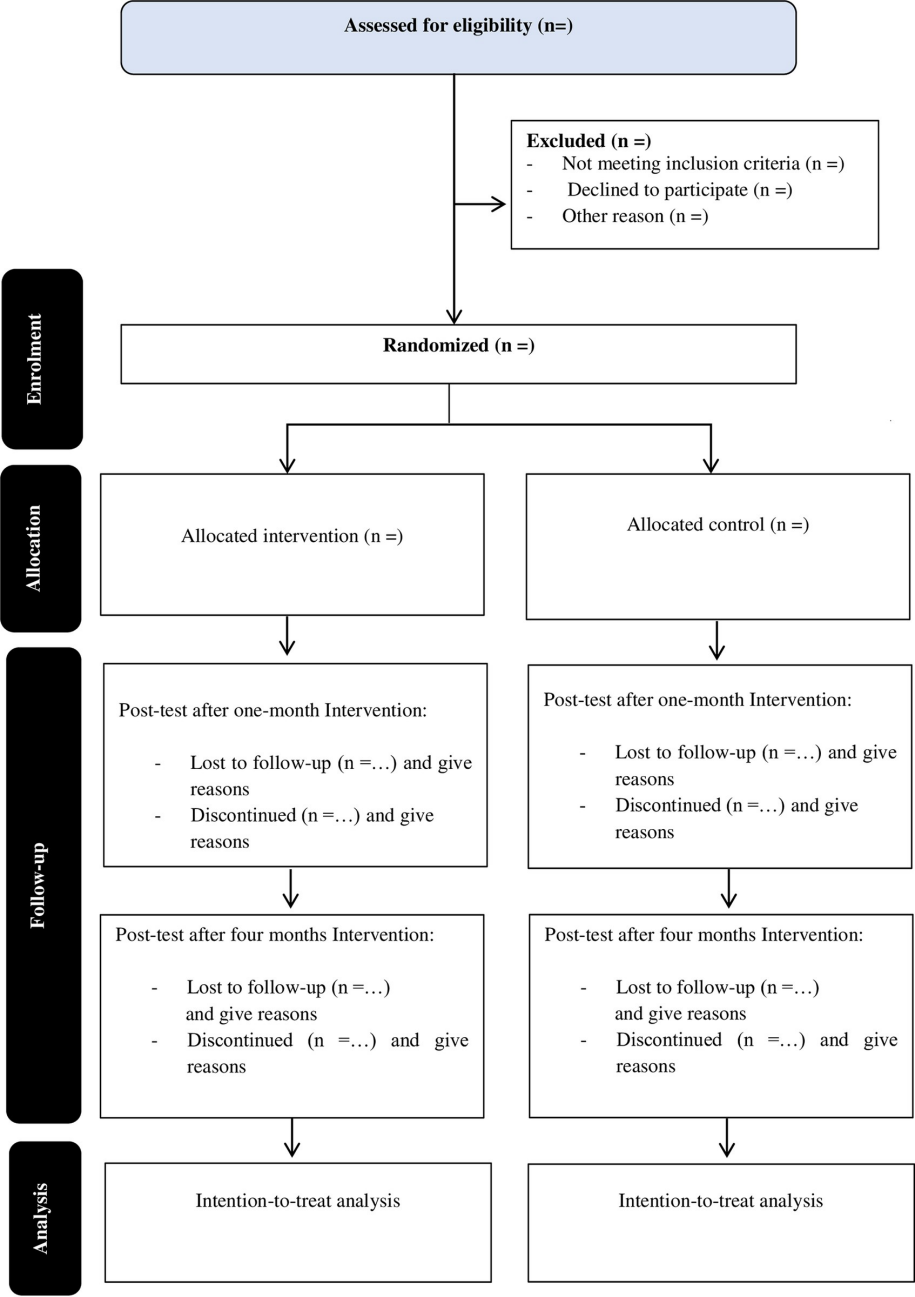
CONSORT flow diagram of the study. Adopted from [24].

*Study principles*. This study protocol reports based on the Study Protocol Items: Recommendations for Interventional Trials (SPIRIT) 2013 Statement [23]. It also complies with the Consolidated Standards of Reporting Trials (CONSORT) statement. Fig 3 below illustrates the Consolidated Standards of Reporting Trials (CONSORT) flowchart [24].

*Randomisation*. Randomisation will be maintained throughout the entire study using sealed envelopes labelled with sequential numbers. These opaque envelopes contain the treatment allocation cards prepared before the trial. An independent researcher will print out random allocation cards based on computer-generated random numbers. The treatment allocation cards will be folded several times before being placed in the envelope. The randomisation sequence will be created using online software [25], with a 1:1 allocation using random block sizes of 4 and 6.

*Allocation concealment mechanism*. A statistician will be enlisted to ensure proper allocation concealment by producing the list of allocation sequences using special block randomisation software. Every respondent will be assigned a unique code placed in a sealed opaque envelope. The research assistant will open the envelopes and assign the respondents to the intervention or control group based on the code list produced by the software. The envelopes will only be opened after the nurse has selected the respondent, obtained informed consent, and collected baseline data to avoid individual-level bias. Hence, outcome measures are collected. The intervention and control groups will be followed for an equal period of 4 months.

*Intervention*. The intervention group will be exposed to an educational intervention on TB treatment adherence. The educational material in the intervention is based on the Health Belief Model (HBM). The educational module includes two main messages of threat and efficacy aimed at enhancing or motivating the patients to engage in danger control through better adherence to TB medication [17]. In the module, the author will explain how the TB disease is acquired, the risks and consequences related to the behaviour of non-adherence, as well as the benefits of TB medication and treatment adherence.

The module for educational intervention was based on six main constructs, i.e., perceived benefit, perceived barriers, perceived susceptibility, perceived severity, perceived self-efficacy, and cue to action, all of which have been incorporated into the intervention Table 1. After the educational intervention was developed, its face and content validity were reviewed and evaluated by a respiratory specialist in the Abu Anga hospital, two public health experts from Universiti Putra Malaysia (UPM), and one public health expert from the University of Khartoum, Sudan. The experts felt that the educational materials were efficient and commented on the understandability, simplicity, and mode of delivery. After amendments, the education booklet was translated into Arabic by a professional translator to prevent any unclear phrases or grammatical errors. The intervention will be given via four sessions of a two-hour workshop in which the educational modules will be delivered in the form of PowerPoint presentation and handouts. Based on the experience of a Bangladeshi study, the intervention will take about 30 minutes for each session [17]. A discussion for 10 to 15 minutes will be conducted after the presentation of the educational module. During the discussion, the researcher will provide support to the patients with any issues related to their medication non-adherence. The researcher will also encourage the patients to perform the appropriate steps such as using a diary to monitor their medication adherence. To facilitate the discussion, the intervention group will be divided into smaller groups of 10 to 12 participants.

**Table 1:** Overview of the educational intervention on TB treatment adherence.

Sessions	Topics	HBM Construct	Area of target	Intervention
TB infection	Causes and transmission Symptoms and complications Does TB cause death?	Perceived susceptibility Perceived seriousness	Knowledge of TB infection	PowerPoint presentation, booklet, small group discussion
TB screening and prevention	Who is more likely to develop TB disease? Diagnosis and prevention	Perceived susceptibility Perceived seriousness	Knowledge of TB	PowerPoint presentation, booklet, small group discussion
Treatment of TB and Adherence to TB treatment	Medication for TB disease Treatment adherence Tips on how to manage poor TB adherence Barriers to medication adherence	Perceived benefits Perceived barriers Health motivation (Cues to action)	Knowledge of TB treatment Belief in TB treatment adherence	PowerPoint presentation, booklet
Goal Setting and Action Planning	Write action plans and set goals that can motivate treatment adherence. -To motivate patients to explore barriers to TB treatment adherence	Perceived benefits Perceived barriers Motivation (Cues to action Self-confidence	To motivate and increase an individual’s confidence to take action to improve their health condition.	PowerPoint presentation and booklet, handout, Participants to write their goal setting for good treatment adherence

After the educational intervention, a booklet with all the information will be offered to the participants. The booklet will include facts about TB, prevention, and screening of tuberculosis infection, treatment of tuberculosis, adherence to treatment, and TB adherence Action Plan. To avoid contamination between the intervention and the control group, the patients in the intervention group will be informed not to share the contents of the educational intervention with another group. During random allocation, participants who know someone else attending the screening will be excluded. In addition, the researchers will hold treatment sessions at different times, and the waiting time will be reduced so that participants in the intervention and control groups will not meet in the waiting room.

*Intervention group*. The educational module will be given to the participants in the intervention group to remind them about TB medication adherence. There will also be recommendations on how to manage poor medication adherence. The research team will also send monthly messages to the intervention group to remind them to adhere to their medications.

*Control group*. The control group members will receive the same routine standard treatment care as the intervention group, but will not receive any education intervention during the study. Instead, they will be provided with similar educational modules with materials and knowledge on TB treatment adherence when the study concludes.

The same set of the questionnaire at baseline, one-month, and four-month after the intervention will be administered for both groups.

#### Outcome measures

*Intervention fidelity*. Several strategies will be employed to ensure that the educational intervention is delivered faithfully. Firstly, the intervention delivery will be provided in such a way as to preserve its consistency while at the same time making sure that the participants are encouraged and reinforced in their compliance with the intervention. Consistency in the delivery of intervention is also ensured by dividing the intervention group into smaller groups. The educational intervention will be independently conducted by the researcher for each of the smaller groups. Uniform delivery of the education intervention for all subgroups can be achieved by adhering to a strict protocol. During the sessions, instant feedback can be provided to the participants by the researcher who plays the part of an observer. Intervention fidelity is also enhanced by sending regular short text messages to keep the participants’ spirits up and to ensure that they will not lapse in TB treatment adherence.

*Participant privacy*. Honesty is particularly stressed to the participants to obtain sincere responses. The participants will be informed that the questionnaires will not contain any identifying information and they will only be tagged with a unique number for identifying them later.

#### Study outcomes

*Primary outcome*. The primary outcome of interest in this study is the treatment adherence for TB medications. The data will be collected using the Medication Adherence Rating Scale (MARS), a 10-item self-reporting instrument that describes three dimensions: medication adherence behaviours (items 1–4), attitude toward taking medication (items 5–8), as well as negative side effects and attitudes to medication (items measured 9–10) [26].

*Secondary outcomes*. The secondary outcome variables in this study include:

#### Knowledge of Tuberculosis

Patients’ TB disease and treatment knowledge will be assessed using an adapted questionnaire that consists of 15 items including causes of TB, symptoms of TB, TB transmission, TB medication, and medication adherence. Permission to use the validated questionnaire from [17] has been obtained by the researchers. A nominal scale of “correct”, “incorrect”, and “I do not know” is used to measure the responses. Each correct answer is given one point while zero point is given for incorrect or “I don’t know” response. The score will range from 0 to 15.

#### Tuberculosis health belief

Next, the perception of health belief among the TB patients will be assessed using the same questionnaire [17]. Based on the six HBM domains. The modified questionnaire consists of 30 questions made up of self-reported measurement scales that represent perceived susceptibility to TB infection (three items), perceived seriousness of TB infection (six items), perceived benefits of TB medication adherence (five items), perceived barriers for TB medication adherence (eight items), confidence in one’s ability to adhere to the medications (four items), and health motivation or cue to action (four items). All items are scored on a Five-Point-Likert scale, i.e., strongly disagree (1 point), disagree (2 points), neutral (3 points), agree (4 points), and strongly agree (5 points). The score for each question will be summed up to determine the total scores. Higher scores indicate greater feelings related to that construct. All scales are positively related to screening behaviours except for barriers, which are negatively associated.

#### Quality of life

The abbreviated version of the World Health Organisation quality of life Questionnaire (WHOQoL-Bref) [27], that consists of 24 items will be used for assessing the participants’ quality of life perceptions in four domains, namely physical health, psychological health, social relationships, and environment within the previous 2 weeks.

With two other items being related to overall quality of life and general health. Scores in the domains are then converted into a linear scale from 0 to 100.

Other outcomes

#### Participantsʹ information

The sociodemographic profiles of the respondents will be assessed using five questions in the questionnaire (age, gender, marital status, educational level, and employment status).

#### Questionnaire and study module translation

The English version of the questionnaires used in this study will be translated using the forward translation and back-translation process. First, all questions are translated from English to Arabic according to the WHO process of translation and adaptation of instruments [28]. Then, the forward translation of the original questionnaire from English to Arabic will be performed by a certified linguistic translator (native speaker of Arabic and expert in English). Following that, back translation from Arabic to English will be done by another independent, qualified, and professional translator (native speaker of Arabic and expert in English). Finally, the back-translated English version is compared with the original questionnaire by another English language translator to ensure no discrepancy between items in the original and translated versions. The researcher then compares the translated version of the module and questionnaire with the original version to decide the accuracy of the final version of the module and questionnaire.

Subsequently, the translated module will be tried out on seven TB patients. It will also be face validated among ten TB patients. All these patients will be excluded in the subsequent randomised controlled trial (RCT). The participants will be asked individually about the clarity and comprehensibility of the questions. Phrases deemed unsuitable and/or hard to understand will then be modified accordingly. Correspondingly, the final version will be endorsed for use in the RCT.

### Statistical analysis

The data will be analysed using IBM SPSS Software 25.0 (SPSS Inc. Chicago, United States). Note that any missing data will be managed by adopting the intention‐to‐treat analysis concept. The continuous variables will be checked for normality prior to analysis. The value of p < 0.05 is set as the alpha level of significance. Baseline data will be outlined with descriptive analysis. For continuous variables, mean, standard deviation, median, and range will be used; frequency and percentage will be used for categorical variables. For the comparison of categorical data between groups, the chi‐square test will be used to compare the differences between the two groups. As for continuous data, an independent sample t‐test will be used. For the primary and secondary interests of analysis, multivariable regression model analysis will be employed to adjust confounding factors in the statistical effect. In addition, Cochran’s Q test will be used to assess the changes in categorical data in both groups over time. In contrast, continuous variables that change over time will be identified using one‐way repeated measures (ANOVA). After testing for assumptions and outliers, between-groups (intervention vs control), the main effect and interaction between and within the two groups over time will be tested using generalised estimating equations.

## Results

The trial findings will be reported based on the Consolidated Standard of Reporting Trials (CONSORT) guidelines. In addition, the research outcomes will be published in an international peer-reviewed open-access journal. The principal investigator is responsible for disseminating the findings to Health facilities, Abu Anga hospital, the Ministry of Health (MOH), Universiti Putra Malaysia, and national and international conferences.

## Discussion

This study protocol outlined the use of a module to examine the effects of tuberculosis (TB) educational intervention on the adherence to TB treatment, knowledge, quality of life (QOL), and health beliefs of Sudanese TB patients in Khartoum state, Sudan using randomised controlled trial (RCT) design. Activities in the intervention have been formulated to be integrated into the routine practices of TB care to foster strong local ownership. A two-way interactive process between patients and physicians is fundamental to ensure adherence. There are many projected benefits from the RCT. It can generate information on the determinants of adherence to TB treatment and evaluate the barriers to reducing non-adherence. Non-adherence is the main challenge in the TB control program globally. As such, information from this RCT is vital to minimise treatment costs, guide policy direction, and improve patients’ QOL. The study findings can be implemented in tandem with the existing TB treatment strategy, such as Directly Observed Therapy (DOT).

To reduce the risk of non-adherence to TB treatment, healthcare workers must be trained to check patients’ adherence during home visits. A few limitations are likely to arise from the RCT. Firstly, the healthcare providers cannot be blinded, which may introduce performance bias. While reporting bias is also possible, it can be minimised by objectively and prospectively collecting the adherence history of patients. We will also ensure that the health workers provide equal care to patients in both groups, whereby the control group will also receive the educational module after the study.

In summary, this RCT will be useful in investigating the impact of TB educational intervention among Sudanese TB patients in Khartoum state, Sudan, especially on their adherence to TB treatment, TB knowledge, QOL, and health beliefs using an RCT design. The two objectives of this study are 1) developing an educational intervention based on the Health Belief Model (HBM) for TB treatment adherence, knowledge, life quality, and beliefs; assessing the effectiveness of the educational intervention on adherence to TB treatment, knowledge, QOL, and beliefs among Sudanese patients in Khartoum state, Sudan, at different time points (baseline, 1 month, and 4 months after intervention).

## Supporting information

S1 Checklist SPIRIT 2013 checklist: Recommended items to address in a clinical trial protocol and related documents. (DOC)

S1 File Ethical approval letter. (DOCX)

S2 File Protocol approved by ethics. (DOCX)
