# Supplementary material for: Effectiveness of education intervention of tuberculosis treatment adherence in Khartoum State: A study protocol for a randomized control trial
Source: PLoS One. 2022 Nov 28;17(11):e0277888. doi: 10.1371/journal.pone.0277888 (PMC9704653; doi:10.1371/journal.pone.0277888)
Supplement: S2 File — (DOCX) [file pone.0277888.s003.docx]

**Overview**

In Sudan, tuberculosis (TB) is a public health primary concern. The national TB program is used by the Government in the effort of involving other sectors in control efforts, detecting cases, and providing care to patients.

Apart from setting and regimen-specific factors, in Sudan adherence to tuberculosis medications is also known to be affected by a wide range of behavioral, financial, and patient-specific demographic factors .Nevertheless, majority of these factors, household income for example, could not be easily altered by healthcare providers. Moreover, it is known for TB patients to contemplate on defaulting numerous times during the course of their treatment, the intensity of their motivation to complete their regimen fluctuating. There is a higher possibility for the development of drug resistance and for the transmission of TB among patients who default in contrast to those who adhere. The Directly observed treatment, short course (DOTS) strategy has been adopted by Sudan since 1993. However, failure to adhere to TB treatment continued among some patients that eventually resulted in their default of the treatment before completing it, in spite of the health authorities’ efforts in addressing the issue.

The intervention will be held in 2 hours as a 1-day workshop, and the educational modules will be used in four sessions in the form of PowerPoint presentations and handouts. The time regimen sessions of the intervention will be set for 30 minutes each . Then a discussion will be conducted for 10 to 15 minutes after the presentation of the educational module. During the discussion, the researcher supported the patients and helped them to solve their medication non-adherence. The researcher also encouraged the patients to act correctly like using a diary to monitor their medication adherence. The researcher will divided the intervention group into groups of 10–12 participants. After the educational intervention was completed, the participants will be given copies of a booklet that contained all the information provided in the educational intervention. The content of the booklet include facts about tuberculosis, prevention and screening of tuberculosis infection, treatment of tuberculosis and adherence to treatment, and TB adherence action plan. The educational module will be given to the participants in the intervention group to remind them about TB medication adherence and to instruct them on how to improve poor medication adherence. The research team also will send monthly messages to the intervention group to remind them to adhere to their medications.

**Background**

Tuberculosis is a single-agent infectious disease, which is the major cause of death around the world. In humans it is caused by Mycobacterium Tuberculosis complex, and in most cases is caused by Mycobacterium Tuberculosis C. In 2015, there were an estimated 10.4 million new (incidents) TB cases worldwide, of which 5.9 million (56%) were among men, 3.5 million (34%) among women and 1.0 million (10%) among children (1). In addition Tuberculosis (TB) is infectious disease that normally infects the lungs (pulmonary TB) and is becomes contagious when bacteria are expelled into the air by people who have been infected with pulmonary TB. However, it is also capable of affecting other parts of the body (extra pulmonary TB) (2).

With Tubercle bacilli infecting roughly one third of the population across the globe, who are at risk of developing the active disease, TB has thus become a public health problem worldwide (2). In 2017, TB was responsible for 1.6 million deaths in the world, and in particular in the developing countries (3).

Tuberculosis (TB) remains a major global public health problem despite the fact that the causative organism has been known for more than 100 years, and highly effective medication and vaccines have been available for decades (4).

In addition Tuberculosis (TB)-HIV co infection is a public health problem of global importance according to the World Health Organization, of all new TB cases, 1.2 million (11%) is accounted by those with HIV infection. In 2019, 10 million people developed tuberculosis (TB) and approximately 1.4 million people died from TB related illnesses worldwide (5). Although cumulative TB incidence reduced approximately 9% from 2015 to 2019, this less than half of the 20% reduction required to meet the World Health Organization (WHO)‘s End TB Strategy milestone between 2015 and 2020 (6)

The World Health Organization (WHO) has recommended the DOT (Directly Observed Therapy) strategy, which has proven to be effective in the improvement of anti-TB treatment adherence, with its key element where health workers or family members are required to directly observe TB patients while taking their medicine (1). Under the standard anti-TB treatment, the new patients are required to be on a drug combination every other day for 6 months and 8 months for retreatment patients. The duration for tuberculosis treatment is six months of minimum treatment for drug-responsive tuberculosis, and between 18 to 24 months for multidrug-resistant tuberculosis (MDR-TB), therefore the duration of TB treatment is long. (7).

Due to the high rate of patients defaulting TB treatment, it is therefore essential that the risk factors that lead to this treatment default to be identified. A previous controlled intervention pilot study conducted in the state of Khartoum that used mobile phone to improve TB treatment adherence concluded that patients in the intervention group had higher documented cure rate (78.4%), better knowledge compared to the control group, and lower default rate (6.8%) (8)

Treatment interruption may result due to such a lengthy and strict regimen that poses a challenge for TB patients in adhering to their prescribed treatment (9). Due to the risk of failure in treatment, development of drug resistance, and prolonged transmission of the disease, non-adherence to tuberculosis (TB) treatment has become a challenge. As suggested by evidence, besides total interruption of treatment (default), intermittent interruption is another concern since treatment non-adherence can result in poor treatment outcomes (10 ). In some resource-limited countries, the rate of DOT coverage is still low and not much increase (9). The non-success of treatment and the recurrence of disease are mostly caused by non-adherence that can result in death, transmission, , drug resistance, and prolonged infection. Stigmatization, financial burden of TB treatment, long duration of treatment, and adverse reaction to the drugs during treatment, are the contributing factors of treatment non-adherence and unsuccessful outcomes of treatment (7).

.

Apart from pharmacological and immunopathological impacts, non-adherence to treatment or intermittent treatment continues to be a significant reason for the development of Multidrug Resistant and Extensively Drug Resistant TB (MDR-TB and XDR-TB) bacilli strain (11). Based on the latest report by the World Health Organization (WHO), there was 3.6% of global predominance/widespread of MDR-TB amidst new TB cases and 20.2% amidst previously (formerly/already) treated TB cases OR amidst cases of TB that have been treated before (12). Studies have shown that adherence to TB-treatment is influenced by a number of factors including perceived stigma, psychological distress, knowledge regarding TB and its treatment, distance to the nearest health facility, , perception concerning disease and its treatment, , , economic status, and change of residence (13).

Poor adherence to treatment and failure to follow up raise the level of mortality, morbidity, and the risk drug resistance development, which can result in the prolonged spread of TB (14).

Apart from reducing the ongoing spread of TB and the development of drug resistance, tuberculosis treatment adherence also increases the chance of cure. According to the WHO (2015), to encourage TB treatment adherence, it is recommended for DOT to be used, which frequently requires enablers, through a patient-centered technique , (15).

A significant association between non-adherence to anti-TB treatment has been discovered in previous studies with patient-related factors, which include recovering, , lack of knowledge on benefits of treatment course completion, male gender, running out of drugs at home, use of herbal medication, distance to the health facility, HIV sero positivity, stigmatization, abuse of alcohol, and forgetfulness ( 16).

In 2013, WHO reported that there were 108 cases per 100,000 population of TB notification rate in Sudan (17).

With an estimated 1.8% of tuberculosis (TB) infection risk yearly, that which results in 90/100,000 incidence of smear positive cases, Sudan is therefore one of the Eastern Mediterranean Region countries with a high prevalence of TB (18).

The aspects of control involved in Sudan’s national tuberculosis control program include, among others, the reduction of risk for others and the care and management of patients. However, the performance of tuberculosis control are undesirably affected by various challenges that include, but are not restricted to; chronic debilitating diseases and HIV infection, poverty, overcrowding, poor nutrition, internal displacement, and ongoing civil war (19).

In the state of Khartoum, Sudan, a previous case control study found that 14% of the TB patients were not adhere to treatment Besides that, the default of TB treatment is associated with several factors, including: distance to health centre (over 5 kilometres), employment, level of education (illiterate), non-existence of family support, and type of residential area (village). However, no statistical relationship was found between TB treatment default in this study and the traditional factors assumed to be connected to the default (for instance family income, age, travelling (cost), family size, religion, and size of house (20).

To monitor patients’ adherence to medication, the guideline for TB treatment as recommended by the World Health Organization (WHO) is the use of the Directly Observed Treatment Short Course (DOTS) strategy. Included in this strategy are the treatment of TB using a six-month regimen of standardized rifampicin for new TB cases, and eight-month regimen for retreatment cases. The sequences of Non-adherence to treatment for tuberculosis has severe human, economic and social costs. Interrupted treatment may reduce treatment efficacy and cause drug resistance resulting in increased morbidity and mortality and further infections (21).

Numerous interventions have been formed and executed in decreasing the level of non-adherence worldwide. For example, an intervention that focused on the empowerment of the specialists and the patients based on the improved TB adherence model proved to have a positive effect on the adherence of TB treatment ( 22). Moreover, previous study conducted among multiple drug resistance tuberculosis patients (MDR-TB) in east Kazakhstan to assess the effects of the patient support program on patient default rates, all patients received the psychosocial support (PSS) program, showed improved adherence to TB treatment, as study concluded that among patients included in the PSS program, no treatment default was observed and only one patient missed doses of treatment (23).

The health belief model (HBM) is recommended since it can be effectively and beneficially used to recognize, describe and predict individual health behavior, which include patients’ adherence to treatment as a health practice (24). This psychological model consists of six domains, namely, perceived barrier, perceived benefit, perceived self-efficacy, and cue to action, perceived susceptibility, and perceived severity. TB patients undergoing treatment, based on the HBM concept, have the likelihood of adhering to their medical regimen subject to certain set of beliefs. First, it is essential for patients to have basic knowledge regarding health and motivation to stay unaffected of TB. Second, it is necessary for them to believe the fatality of the disease, and to believe in the severe health and medical consequences of treatment non-adherence. Third, it is essential for patients to be convinced about the effectiveness of their present TB treatment - that the control of the heir TB infection is possible to be obtained at an acceptable tolerable psychological or tangible social barrier, and that the benefits outweigh the barriers Fourth, the existence of “cue to action”, an external or internal stimulus that triggers patients’ health behavior, for example in taking medication. Finally, the belief of self-efficacy in the patients’ that regular follow-up treatment is to be sustained until the final treatment is completed (25).

**Research Hypothesis**

1. The level of TB treatment is statistically significant higher in the intervention group than in the control group at one month and four months after the intervention.

2. The mean of Health Beliefs Model (HBM) domains is statistically significant higher in the intervention group than in the control group at one month and four months after the intervention

3. The mean of quality of life is statistically significant higher in the intervention group than in the control group at one month and four months after the intervention.

4. The mean of tuberculosis knowledge is statistically significant higher between the intervention group and control group at one month and four months after the intervention

**General Objective**

To develop, implement and evaluate an educational intervention to enhance TB treatment adherence among Sudanese patients with tuberculosis infection based on Health Beliefs Model (HBM).

## **Specific Objectives**

- 1. To develop and implement an educational module in improving tuberculosis treatment adherence among tuberculosis patients in the state of Khartoum, Sudan.
  2. To compare the mean of tuberculosis treatment adherence within and between the intervention group and control group at base line, one month, and four months after the intervention.
  3. To establish the effect of educational intervention on tuberculosis treatment adherence for the intervention group and control group at baseline, one month, and four months after the intervention after controlling for covariates.

1. To compare the mean difference within and between the intervention group and control group on the Health Beliefs Model (HBM ) domains (perceived susceptibility, perceived severity, perceived benefits, perceived barriers, perceived self-efficacy, cue to action) at baseline, one month, and four months after the intervention after controlling for covariates.
2. To compare the Quality of Life (QOL) within and between the intervention group and control group at baseline, one month, and four months after the intervention after controlling for covariates.
3. 6. To compare the mean difference within and between the intervention group and control group on tuberculosis knowledge at baseline, one month, and four months after the intervention after controlling for covariates

## **Methods:**

## **Research Design.**

This is Randomized Control Trial (RCT) which is a parallel trial with one control group and one intervention group will be conducted among pulmonary tuberculosis patients who have been diagnosed with TB based on the National Treatment Guideline under DOTS strategy. The participating patients will not aware of group assignment (single blind). The education material will be given to the intervention group after baseline data was collected and the control group received the education material at the end of the study. Assessments of both groups will be carried out at baseline, 1 month, and 4 months post-intervention.

## **Methods**

The randomization will be maintained throughout the entire study. This will be achieved using opaque sealed envelopes that were numbered in a sequence and contained the treatment allocation cards, which were prepared prior to the study. First, an independent researcher will make random allocation cards using computer-generated random numbers, the treatment allocation cards will be printed out for each one and put in an envelope after being folded several times. The randomization sequence will be created using the web page at (Sealed Envelope. Create a Randomisation List, 2001) with a 1:1 allocation using random block sizes of 4 and 6 made by the researcher. The block randomization will be used to ensure an equal allocation of participants to each group (Efird, 2011). The allocation sequence will be kept securely in a separate location. The concealed allocation will be attained using opaque envelopes that will be numbered sequentially and then sealed after inserting into them the treatment allocation cards that had been made prior to the study. The numbers will be written sequentially on the front of the sealed envelope to be distributed according to patient’s attendance (e.g. 1, 2 and 3). These numbers will be used to identify participants on the questionnaire and to maintain confidentiality.

After the inclusion of the participants in the trial and the baseline assessment had been taken, a nurse in the tuberculosis clinic who didn’t know anything about the research will be responsible for opening the envelopes in sequence and assigning participants to study group. The intervention will be implemented and designed to improve tuberculosis adherence, TB knowledge, TB quality of life, and to modify beliefs related to tuberculosis medication adherence. . The research team also will send monthly messages to the intervention group to remind them to adhere to their medications. During the period of the study, the control group’s participants will not receive any education. At the study’s end, however, they wll be provided the same educational materials on TB treatment adherence. The participants in this group also will required to answer the same questionnaire set at baseline, 1 month, and 4 months after the intervention.

## **Subjects and Recruitment.**

18 years or older tuberculosis patients on full course of TB treatment under Directly observed treatment, short course ( DOTS) for one or two months prior to the study , capable of physically and mentally providing informed consent. and can follow intervention will be chosen to participate in the study The researcher will inform the respiratory specialist and medical doctors in the tuberculosis clinics about the study objectives and the eligibility criteria to facilitate patient selection for the Randomized controlled trial (RCT ) . After the tuberculosis patients complete their appointment with the physician in the respiratory clinic, the nurse will direct the tuberculosis patients who fulfil the inclusion criteria to the researcher. The researcher then explains the purpose and benefits of this study. Tuberculosis patients who agree to join will be asked to complete a written consent form to the relevant authority in Abu Anga hospital before they are being asked to answer the questionnaire. After obtaining the baseline assessment, a nurse in the tuberculosis clinic will open a sealed envelope to assign the participants to either the intervention or the control groups. Subsequently, the intervention group will be given the tuberculosis educational intervention. For the post-intervention and follow‐up assessments, the participants will be asked to fill in the same questionnaire without the section on personal information. Participant.

## **Outcome measures.**

Data will be collected at three points of time during the study (baseline, 1-month post-intervention, and 4-month follow up) using a previously validated questionnaire.

**Time frame & information collection at the three time points**

|  | | | | |
| --- | --- | --- | --- | --- |
| Information | Time | | | |
|  | Baseline 1 month | | 4months |  |
| Personal information of participants |  |  |  |  |
| Knowledge of TB |  |  |  |  |
| Health Belief Model (HBM) |  |  |  |  |
| Treatment adherence and Quality of life |  |  |  |  |

At baseline, descriptive characteristics including information about the age, gender, marital status, educational level, and employment status of participants will be collected, the other out comes will be collected base line , one month and four month after the intervention this incude :

treatment adherence ,according to the World Health Organization, treatment adherence is “the extent to which a person's behavior taking medication, following a diet, and/or executing lifestyle changes corresponds with the agreed recommendations from a healthcare provider” (WHO, 2003).

Quality of Life is defined by the WHO as an individual's perception of their position in life in the context of the culture and value systems in which they live and in relation to their goals, expectations, standards and concerns (WHO, 1997).

Patients’ TB knowledge was assessed using a designed structured questionnaire. The self-administrated questionnaire used in the current study consists of 15 items including causes of TB, symptoms of TB, TB transmission, TB medication and medication adherence. **Health Belief Model**

To enhance the effectiveness of the health educational program on TB treatment adherence, the program was grounded in the six constructs of the HBM. According to Glanz K (2008), the six constructs are defined as follows

**Perceived Susceptibility:** “tuberculosis patient’s opinion of chances of getting both medication susceptible and resistant types of TB”.

1. **Perceived Severity:** “tuberculosis patient’s opinion of how serious both medication susceptible and resistant types of TB are and their consequences”.
2. **Perceived Benefit:** “tuberculosis patient’s belief in the efficacy of TB medication and on the importance of treatment adherence to reduce risk or seriousness of the disease”.
3. **Perceived Barriers:** “TB patient’s opinion of the tangible and psychological costs of treatment adherence”.
4. **Cue to Action Health Motivation:** “Factors that motivate or activate TB patient’s readiness to adhere to his/her treatment”.

**Self-efficacy:** “Confidence in TB patient’s ability to follow his/her treatment correctly until complete”.

**CONSORT Flow Diagram of the Study as Adopted from (21)**


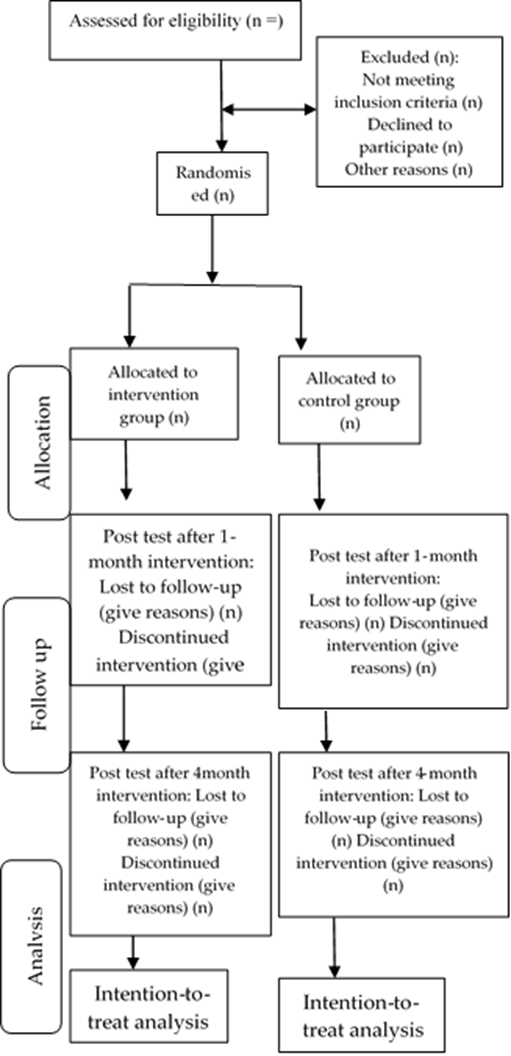


**Intervention**

The educational material in this study will be based on the health belief model. The researcher will develope an educational module by introducing two messages of threat and efficacy to enhance or motivate the patients to carry out danger control through better adherence to TB medication (Tola HH et al., 2016). In the module the author will explaine TB disease acquisition, the risks and harm related to non-adherence behavior, benefits of TB medication and treatment adherence. The educational intervention module used six constructs of perceived benefit, perceived barriers, perceived susceptibility, perceived severity, perceived self-efficacy, perceived threat and cue to action, which were incorporated into the intervention, This intervention will be implemented and designed to improve tuberculosis adherence, TB knowledge, TB quality of life, and to modify beliefs related to tuberculosis medication adherence.

The intervention will be held in 2 hours as a 1-day workshop, and the educational modules were used in four sessions in the form of PowerPoint presentations and handouts. The time regimen of the intervention will be set based on experience from Bangladesh; the sessions will be broken into sets of 30 minutes each (Tola HH et al., 2016). Then a discussion will be conducted for 10 to 15 minutes after the presentation of the educational module. During the discussion, the researcher will support the patients and helped them to solve their medication non-adherence. The researcher also will encourage the patients to act correctly like using a diary to monitor their medication adherence. The researcher will divide the intervention group into groups of 10–12 participants.

## **Treatment Group**

As mentioned before the treatment group will receive the educational intervention module, after the educational intervention will be completed, the participants will be given copies of a booklet that contained all the information provided in the educational intervention. The content of the booklet will include facts about tuberculosis, prevention and screening of tuberculosis infection, treatment of tuberculosis and adherence to treatment, and TB adherence action plan. The educational module will be given to the participants in the intervention group to remind them about TB medication adherence and to instruct them on how to improve poor medication adherence. The research team also will send monthly messages to the intervention group to remind them to adhere to their medications.

**Control Group**

During the period of the study, the control group’s participants will not receive any education. At the study’s end, however, they will be provided the same educational materials on TB treatment adherence. The participants in this group also will be required to answer the same questionnaire set at baseline, 1 month, and 4 months after the intervention.

## **Data Analysis**

Before data analysis began, values of all variables will be checked for errors, then corrections will be made for all scores that fell out of possible ranges of values through refereeing into the original data. Data will be also cleaned and explored for missing values.

- Descriptive information will be determined to describe the socio-demographic data. Data will be checked for normality and homogeneity distribution before assessment of association by using Kolmogorov Smirnov or Shapiro-Wilk. The data will be analysed using a Statistical Package for Social Science (SPSS) version 22. The level of significance will set at alpha to equal .05.
- Descriptive analysis will be firstly carried out to examine the demographic status of participants in this study. The raw data will be checked first for normal distribution for all variables in the analysis. For categorical data, chi-square will be used whilst, for continuous data, t-test will be used to compare between intervention and control group at base line.
- To assess the changes within both groups, the one-way repeated measurement ANOVA will be used for continuous and normally distributed variables was used for continuous and normally distributed variables such as; knowledge of TB, the six domains of health believe model, and quality of life to compare the mean difference within intervention and within control groups at baseline, 1-months and 4-months.
- Generalized linear mixed model will be done as multilevel modelling for the adjustment which will be used for the cluster (strata) effect in estimating the standard errors of the parameter estimates.

The fixed and random effects model will be used for estimation of slopes and intercepts, because the researcher will collect data from different hospital, “hospital” will be thought of as random.

- Baseline outcomes will be compared to see whether the two study arms (ie, control and treatments groups) are similar before the intervention and there are no significant differences observed between the two groups.
- Intervention effects (intervention minus control) will be assessed using generalized estimating equations (GEE) to account for repeated measures across Time 1, Time 2 and Time 3 on outcome variables.
- Study hypotheses will be tested using an intention-to-treat analysis, where by all consenting patients who will randomize to each group will be included in the analysis.

# **Sample Size**

The sample size is calculated using the standard sample size estimate for an individually randomised design. The formula to calculate the difference between two population proportions will be used (power = 0.80, alpha = 0.05 two‐sided, P1 = 0.82, P2 = 0.69) [19].

$$N=\frac{\{1.96\begin{matrix} \sqrt{2\times0.33(1-0.33)} & + 0.842\sqrt{0.82\left( 1-0.82 \right)+o69(1-0.69)} & {\}}^{2} \end{matrix}}{{(0.82-0.69)}^{2}}$$

N = 171 for each group

By taking into account a 10% attrition rate, a total sample size of 188 (171 + 17 = 188) participants will be required for each group, giving a total sample size of 376 participants

## **Study Sites**

The study will be based at the Abu Anga regional referral hospital in Omdurman city, Khartoum state, Sudan, and acts as Sudan’s primary TB specialist hospital. All cases of suspected resistant TB are sent to this facility when confirmation is needed. The hospital also provides healthcare services to the people of Khartoum and neighbouring states, as well as caring for suspected TB cases.

## **References**

1. World Health Organization (WHO). DOTS. Geneva. 2016. Available at:http://who.int/tb/dots/en/. Accessed 22 November 2016.

2.Tola, H. H., Holakouie-Naieni, K., Tesfaye, E., Mansournia, M. A., & Yaseri, M. (2019). Prevalence of tuberculosis treatment non-adherence in Ethiopia: A systematic review and meta-analysis. *International Journal of Tuberculosis and Lung Disease*, *23*(6), 741–749. <https://doi.org/10.5588/ijtld.18.0672>.

3.Iweama, C. N., Agbaje, O. S., Umoke, P. C. I., Igbokwe, C. C., Ozoemena, E. L., Omaka-Amari, N. L., & Idache, B. M. (2021). Nonadherence to tuberculosis treatment and associated factors among patients using directly observed treatment short-course in north-west Nigeria: A cross-sectional study. *SAGE Open Medicine*, *9*, 205031212198949. <https://doi.org/10.1177/2050312121989497>.

4. Fang, X. H., Shen, H. H., Hu, W. Q., Xu, Q. Q., Jun, L., Zhang, Z. P., Kan, X. H., Ma, D. C., & Wu, G. C. (2019). Prevalence of and factors influencing anti-tuberculosis treatment non-adherence among patients with pulmonary tuberculosis: A cross-sectional study in Anhui Province, Eastern China. *Medical Science Monitor*, *25*, 1928–1935. <https://doi.org/10.12659/MSM.913510>.

5.World Health Organization. Tuberculosis. Key facts. Geneva; 2020. https:// www.who.int/news-room/fact-sheets/detail/tuberculosis. Accessed 23 Apr 2021.

6.World Health Organization. Tuberculosis.Glopal tuberculosis report2 2021 available at <file:///C:/Users/USER/Downloads/9789240037021-eng.pdf> accessed on 2nd June 2021.

.

7.Tanimura T, Jaramillo E, Weil D, Raviglione M, Lonnroth K (2014) Financial burden for tuberculosis patients in low- and middle-income countries: a systematic review. Eur Respir J.

8.Ali, A. O. A., & Prins, M. H. (2019). Mobile health to improve adherence to tuberculosis treatment in khartoum state, sudan. *Journal of Public Health in Africa*, *10*(2). https://doi.org/10.4081/jphia.2019.1101.

9. Lei, X., Huang, K., Liu, Q., Jie, Y., & Tang, S. (2016). Are tuberculosis patients adherent to prescribed treatments in China ? Results of a prospective cohort study. *Infectious Diseases of Poverty*, *5*(38), 1–9. <https://doi.org/10.1186/s40249-016-0134-9>.

10. Jakubowiak, W. M., Bogorodskaya, E. M., Borisov, S. E., Danilova, I. D., & Kourbatova, E. V. (2007). Risk factors associated with default among new pulmonary TB patients and social support in six Russian regions. Int J Tuberc Lung Dis, 11(1), 46-53.

.

11.Hirpa S, Medhin G, Girma B, Melese M, Mekonen A, Suarez P.(20013). Determinants of multidrug-resistanttuberculosis in patients who underwent first-line treatment in Addis Ababa: a case control study. BMC Public Health 13(1):1.

12.World Health Organization. (‎2014)‎. Global tuberculosis report 2014. World Health Organization. Available at  <https://apps.who.int/iris/handle/10665/137094> accessed on third of November 2020.

13. Kulkarni P, Akarte SV, Mankeshwar RM, Bhawalkar JS, Banerjee A, Kulkarni A. (2013).Non-Adherence of New Pulmonary Tuberculosis Patients to Anti-Tuberculosis Treatment. Ann Med Heal Sci Res. 3(1):67–74.

.

14. M'Imunya J M, Kredo T, Volmink J. (2012). Patient education and counselling for promoting adherenceto treatment for tuberculosis. Cochrane Database Syst Rev 5: Cd006591. doi: 10.1002/14651858.CD006591.pub2 PMID: 22592714.

15.World Health Organisation. Global tuberculosis report. Geneva: WHO; 2015. . Global Health Observatory Database. World Health Organisation, 2015. At http://apps.who.int/gho/data/?theme=main. Accessed 18 Dec 2016

16.Tesfahuneygn, G., Medhin, G., & Legesse, M. (2015). Adherence to Anti ‑ tuberculosis treatment and treatment outcomes among tuberculosis patients in Alamata District , northeast Ethiopia. *BMC Research Notes*, 1–11. <https://doi.org/10.1186/s13104-015-1452-x>.

17.Banaga, A. S. I., Siddiq, N. K., Alsayed, R. T., Babiker, R., & Elmusharaf, K. (2016). *of Kidney Diseases and Transplantation Renal Data from the Arab World Prevalence and Presentation of Tuberculosis among Hemodialysis*. *27*(5), 992–996.

18.Ahmed Suleiman MM, Sodemann M, Aro AR (2009). Evaluation of tuberculosis control programme in Khartoum State for the year 2006. Scandinavian Journal of Public Health,37(1):101-8.

19. Elmadhoun, W. M., Noor, S. K., Bushara, S. O., Ahmed, E. O., Mustafa, H., Sulaiman, A. A., Almobarak, A. O., & Ahmed, M. H. (2013). *Epidemiology of tuberculosis and evaluation of treatment outcomes in the national tuberculosis control programme , River Nile state , Sudan , 2011 – 2013*. *22*(2), 95–102.

20. Ahmed Osman Ahmed Ali,&, Martin Hendrik Prins. (2016). Patient non adherence to tuberculosis treatment in Sudan: socio demographic factors influencing non adherence to tuberculosis therapy in Khartoum StateThe Pan African Medical Journal, 2(10), 116041.

21.Nglazi, M.D., Bekker, LG., Wood, R. *et al.* Mobile phone text messaging for promoting adherence to anti-tuberculosis treatment: a systematic review. *BMC Infect Dis* **13,**566 (2013). <https://doi.org/10.1186/1471-2334-13-566>.

22.Lee, S., Khan, O. F., Seo, J. H., Kim, D. Y., & Park, K. (2013). Impact of Physician ’ s Education on Adherence to Tuberculosis Treatment for Patients of Low Socioeconomic Status in Bangladesh. *Chonnam Medical Journal*, *49*(1), 27–30.

23.Kulkarni P, Akarte SV, Mankeshwar RM, Bhawalkar JS, Banerjee A, Kulkarni A. (2013).Non-Adherence of New Pulmonary Tuberculosis Patients to Anti-Tuberculosis Treatment. Ann Med Heal Sci Res. 3(1):67–74.

24.Redding CA, Rossi JS, Rossi SR, Velicer WF, Prochaska JO (2000). Health behaviour models. Int Electr J Health Educ (3):180-193.

25.Tola, Habteyes Hailu, Shojaeizadeh, D., Tol, A., Garmaroudi, G., Yekaninejad, M. S., Kebede, A., Ejeta, L. T., Kassa, D., & Klinkenberg, E. (2016a). Psychological and Educational Intervention to Improve Tuberculosis Treatment Adherence in Ethiopia Based on Health Belief Model : A Cluster Randomized Control Trial. *Plose One*, *10*(1371), 1–15. https://doi.org/10.1371/journal.pone.0155147.
