# Supplementary material for: Effectiveness of education intervention of tuberculosis treatment adherence in Khartoum State: A study protocol for a randomized control trial
Source: PLoS One. 2022 Nov 28;17(11):e0277888. doi: 10.1371/journal.pone.0277888 (PMC9704653; doi:10.1371/journal.pone.0277888)
Supplement: S1 File — (DOCX) [file pone.0277888.s002.docx]

Ref. no: UPM/TNCPI/RMC/JKEUPM/1.4.18.2 (JKEUPM)
Date: 2/12/2017


Dear Prof / Dr. / Mr. / Ms.,


**APPLICATION FOR JKEUPM ETHICAL CLEARANCE: APPROVED**

With reference to the above, I am pleased to inform you that your application for ethical clearance for the research project entitled **‘Effectiveness of Educational Intervention on Tuberculosis Treatment Adherence in Khartoum State ’** has been approved.

Please note that the official letter of approval will be issued as soon as possible. However, the ethical clearance is considered effective from the date of this email, and you may now proceed with your research.

**Kindly please remind the ethical approval is required in the case of amendments/ changes to the study documents/ study sites/ study team.**

**Researchers should also complete a Study Final Report upon study completion.** The form can be obtained from the Ethics Committee for Research Involving Human Subjects (JKEUPM) website (<http://www.rmc.upm.edu.my/documentfile>).

If you have any enquiries, please contact Ms. Nursuraya (03-89471605) or Ms. Nor Ellia (03-89471244).

Note: Please use this reference number for any transaction.
 - **JKEUPM (FPSK-P143)2017**

Thank you.

Yours faithfully,

Prof.’ Dr. Zamberi Sekawi

Chair

Ethics Committee for Research Involving Human Subjects

Universiti Putra Malaysia
